# Long Course Hyperbaric Oxygen Stimulates Neurogenesis and Attenuates Inflammation after Ischemic Stroke

**DOI:** 10.1155/2013/512978

**Published:** 2013-02-21

**Authors:** Ying-Sheng Lee, Chung-Ching Chio, Ching-Ping Chang, Liang-Chao Wang, Po-Min Chiang, Kuo-Chi Niu, Kuen-Jer Tsai

**Affiliations:** ^1^Institute of Clinical Medicine, National Cheng Kung University, Tainan 704, Taiwan; ^2^Department of Emergency Medicine, Kaohsiung Municipal Ta-Tung Hospital, Kaohsiung Medical University, Kaohsiung 807, Taiwan; ^3^Department of Surgery, Chi Mei Medical Center, Tainan 710, Taiwan; ^4^Department of Biotechnology, Southern Taiwan University, Tainan 710, Taiwan; ^5^Department of Surgery, National Cheng Kung University, Tainan 704, Taiwan

## Abstract

Several studies have provided evidence with regard to the neuroprotection benefits of hyperbaric oxygen (HBO) therapy in cases of stroke, and HBO also promotes bone marrow stem cells (BMSCs) proliferation and mobilization. This study investigates the influence of HBO therapy on the migration of BMSCs, neurogenesis, gliosis, and inflammation after stroke. Rats that sustained transient middle cerebral artery occlusion (MCAO) were treated with HBO three weeks or two days. The results were examined using a behavior test (modified neurological severity score, mNSS) and immunostaining to evaluate the effects of HBO therapy on migration of BMSCs, neurogenesis, and gliosis, and expression of neurotrophic factors was also evaluated. There was a lower mNSS score in the three-week HBO group when compared with the two-day HBO group. Mobilization of BMSCs to an ischemic area was more improved in long course HBO treatments, suggesting the duration of therapy is crucial for promoting the homing of BMSCs to ischemic brain by HBO therapies. HBO also can stimulate expression of trophic factors and improve neurogenesis and gliosis. These effects may help in neuronal repair after ischemic stroke, and increasing the course of HBO therapy might enhance therapeutic effects on ischemic stroke.

## 1. Introduction

Ischemic stroke is characterized by the interruption of blood flow and oxygen to brain tissues [1]. During focal ischemia, tissue surrounding the ischemic core is called penumbra, which is still viable and is a possible target to be rescued [2]. The only effective treatment approved in clinical practice at present is early thrombolytic therapy and reperfusion. However, most patients with ischemic stroke failed to receive proper management in time. Stroke remains an important cause of death and disability for humans and stroke therapy remains an important health issue today.

Hyperbaric oxygen (HBO) has been used as a primary or adjunctive stroke therapy over years. Mechanism of the neuroprotection of HBO treatment after ischemia was thought to be mediated by improving oxygen supply [3]. A good body of evidence suggests that HBO treatment is neuroprotective. HBO treatment can decrease infarction volume on MRI examination and improve neurological outcome [4]. Hyperbaric oxygen was also found to decrease ischemia-reperfusion injury induced by neutrophil [5]. Researchers have also demonstrated that exposure to HBO will cause rapid mobilization of bone marrow stem cells in humans, and the number of bone marrow stem cells (BMSCs) remains elevated in peripheral blood during the course of HBO treatments [6].

BMSCs transplantation in rats has been shown to improve outcome of various neuronal diseases, such as ischemic stroke [7], spinal cord injury [8], and traumatic brain injury [9]. However, the “homing” of BMSCs is very important in regenerative therapy. In this study, we tested the hypothesis that HBO could promote the mobilization and migration of BMSCs to ischemic brain, attenuating ischemic injury, and improving functional recovery. We evaluated the therapeutic effects of HBO on transient middle cerebral artery occlusion (MCAO). Further we tested the hypothesis that BMSCs would improve neurogenesis, gliosis, neurotrophic factor (brain-derived neurotrophic factor, BDNF; nerve growth factor, NGF; and glial-derived neurotrophic factor, GDNF) level, and the expression of MPO (presenting neutrophil activity).

## 2. Materials and Methods

### 2.1. Animal

**Figure 1:**
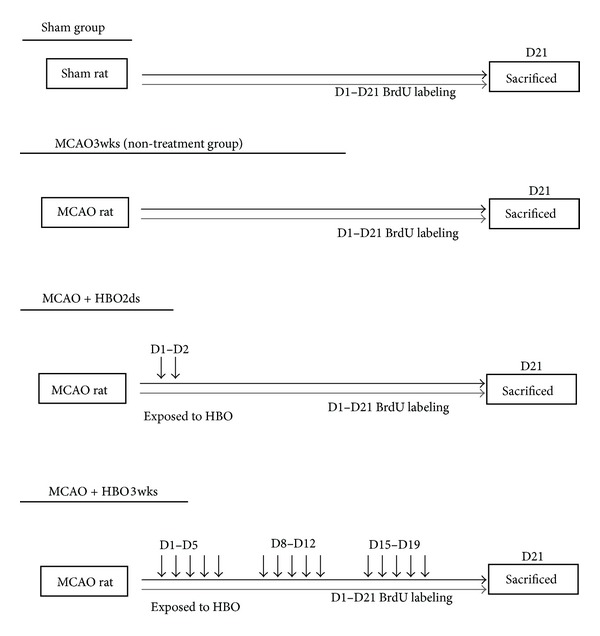
Demonstration of HBO treatment protocol in different groups.

Adult male Sprague-Dawley rats weighing 270 g to 320 g were used in these experiments. They were on a 12-hour light/dark cycle and allowed free access to food and water. All experimental procedures followed the instructions of the Taiwan National Science Council and were approved by the National Cheng Kung University Animal Care and Use Committee with every effort being made to minimize discomfort during the surgery and recovery period. After MCAO insult or Sham procedure, the rats were subjected to hyperbaric oxygen (HBO) therapy or the normal oxygen condition according to group destination. The rats were randomly assigned to one of four groups: (1) Sham group: rats received surgical procedure but without MCAO; (2) nontreatment group: rats sustained MCAO but without HBO treatments; (3) HBO2ds: rats sustained MCAO and received two days HBO treatment course, sacrificed on day 21; (4) HBO3wks: rats sustained MCAO, and received 15-days HBO (5 days/week) course, sacrificed on day 21. The flow charts are represented in Figure 1. Food and water were freely available ad libitum throughout the experimental course.

### 2.2. Middle Cerebral Artery Occlusion

Rats were anesthetized with intraperitoneal (ip) injection of ketamine (2 mg/100 g). Transient middle cerebral artery occlusion [10–12] was induced using the method of intraluminal vascular occlusion. Temperature was continuously monitored with a rectal probe and maintained at 37.0°C with a thermostatically controlled heating pad. Briefly, the right common carotid artery, external carotid artery (ECA), and internal carotid artery (ICA) were exposed. A 4–0 monofilament nylon suture, with its tip rounded by heating near a flame, was advanced from the ECA into the lumen of the ICA until it blocked the origin of the MCA. One hour after MCAO, reperfusion was achieved by withdrawal of the suture until the tip cleared the lumen of the ECA.

### 2.3. HBO Therapy

HBO sessions were conducted as the following procedures. Each rat was put into HBO chamber after reperfusion, then was treated with 100% oxygen at 253 kPa (2.5 atm) for 90 mins. The chamber filled with pure oxygen (100%) was pressurized to 253 kPa at a rate of 51 kPa/min for 90 mins and was terminated at the decompression rate of 20 kPa/min.

### 2.4. Assessing Cerebral Infarction and Functional Outcome

Functional outcome was evaluated using modified neurological severity score (mNSS) [13]. The rats were evaluated by several tests, such as raising rat by tail, placing rat on floor, and beam balance walking, then all test scores were added into the mNSS score (as shown in Table 1). The mNSS evaluations were performed before MCAO and at 1, 7, 14, and 21 days after MCAO. In addition, the triphenyltetrazolium chloride (TTC) staining [14] was used to check the brain infarction size. The colorless TTC is reduced to a red formazan product by dehydrogenases, which are most abundant in mitochondria [15]. Rats were sacrificed at day 3 and day 21. Due to TTC staining is a function test of dehydrogenase enzyme activity and is usually used for early histochemical diagnosis of infraction [16]. Therefore, rats were sacrificed at day 3 to check infraction change by TTC. After documenting mNSS score till day 21, rats were all sacrificed on day 21, then to do immunochemical staining. Under deep anesthesia (Sodium pentobarbital, 100 mg/kg, ip) rats were perfused intracardially with saline. The brain tissue was then removed, immersed in cold saline for 5 min, and sliced into 2.0 mm sections. The brain slices were incubated in 2% TTC dissolved in Phosphate buffered saline (PBS) for 30 min at 37°C and then transferred to 5% formaldehyde solution for fixation.

### 2.5. BrdU Labeling

BrdU (Roche), a thymidine analogue that is incorporated into the DNA of dividing cells during S phase, was used for mitotic labeling. The labeling protocol followed those previously described [17]. A cumulative labeling method was used to examine the population of proliferative cells, with the rat receiving daily intraperitoneal injections of 50 mg/kg BrdU for 15 consecutive days, starting on the first day of HBO. The BrdU^+^ cells in both hemispheres of the hippocampus and cortex were digitally counted with the use of a 20x objective lens with a laser scanning confocal microscope (LSM510; Carl Zeiss MicroImaging, Inc.) via a computer imaging analysis system (Imaging Research). For each animal, 40 coronal sections (each 12 *μ*m thick) throughout the hippocampus and cortex were analyzed. The image analysis was also used to examine the distributions of BrdU^+^ cells with Neu-N and GFAP.

### 2.6. Immunohistochemistry

Animals were allowed to survive for 21 days after MCAO, and at that time animals were sacrificed with urethane (1.5 g/kg, IP). Rat brains were fixed by transcardial perfusion with saline, followed by perfusion and immersion in 4% paraformaldehyde. The brains were removed and then immersed in PBS with 15% and 30% sucrose overnight. The indirect lesion area (the intact area of the ipsilateral hemisphere) was subtracted from the area of the contralateral hemisphere and was calculated. The lesion volume is presented as a volume percentage of the lesion compared with the contralateral hemisphere. 

Single or double immunofluorescence staining was used to identify cells derived from BMSCs. For staining, adjacent 12 *μ*m thick sections were consecutively (1) 2 mol/L HCl-incubated for 30 minutes, (2) rinsed with 0.1 mol/L boric acid (pH 8.5) at room temperature for 10 minutes, (3) incubated overnight with primary antibodies in PBS containing 0.5% normal donkey serum at 4°C, and (4) incubated at room temperature for 1 hour with secondary antibodies. The antibodies were, sequentially, rat monoclonal anti-BrdU (abcam, 1 : 200), rat monoclonal anti-CD34 (Biolegend, 1 : 200), mouse monoclonal anti-NeuN (Millipore, 1 : 200), rabbit polyclonal anti-GFAP (Millipore, 1 : 1000), rabbit polyclone anti-Myeloperoxidase (abcam, 1 : 100), DAPI (Sigma, 1 : 1000), Alexa Flour 488-conjugated goat anti-mouse antibodies, Alexa Flour 488-conjugated donkey anti-rabbit antibodies, Alexa Flour 488-conjugated donkey anti-mouse antibodies, and the Alexa Flour 594-conjugated donkey anti-rat antibodies (Invitrogen). The sections without BrdU were then incubated with DAPI and coverslipped the mounting medium (fluorescent mounting medium; Dako).

### 2.7. Laser Scanning Confocal Microscopy and Cytometry

Colocalization of BrdU with neuronal marker was conducted by laser scanning confocal microscopy (LSCM) with the use of a Bio-Rad MRC 1024 (argon and krypton) laser scanning confocal imaging system mounted onto a Zeiss microscope (Bio-Rad). For immunofluorescence double-labeled coronal sections, green (FITC for NeuN and GFAP) and red cyanine-5.18 (Cy5 for BrdU) fluorochromes on the sections were excited by a laser beam at 488 nm and 647 nm; emissions were sequentially acquired with 2 separate photomultiplier tubes through 522 nm and 680 nm emission filters, respectively. Interested cells were counted with tissue cytometry using TissueQuest software.

### 2.8. Reverse Transcription PCR

Hippocampi from Sham, MCAO, HBO2ds, and HBO3wks were dissected. Total RNAs were isolated using TRIzol reagent (Invitrogen) according to manufacturer's instructions. Reverse transcription of equal amounts of total RNA were carried out using Superscript II First-Strand Synthesis kit (Invitrogen) according to the manufacturer's instructions. Obtained cDNA were amplified using the following primers: for BDNF, 5′-CAGTGGACATGTCCGGTGGGACGGTC-3′ and 3′-TTCTTGGCAACGGCAACAAACCACAAC-5′; for GDNF, 5′-AGGGGCAAAAATCGGGGGTG-3′ and 3′-GCATGCATCCACGACTCGGA-5′; and for GAPDH, 5′-GACCCCTTCATTGACCTCAAC-3′ and 3′-TCTTACTCCTTGGAGGCCATG-5′.

### 2.9. Statistical Analysis

Results are expressed as the mean ± SE for three or more independent experiments. To compare data, we used the ANOVA test. A value of *P* < 0.05 was considered to be statistically significant.

## 3. Results

### 3.1. HBO Improved Functional Outcome and Decreased Infarction Size

**Figure 2:**
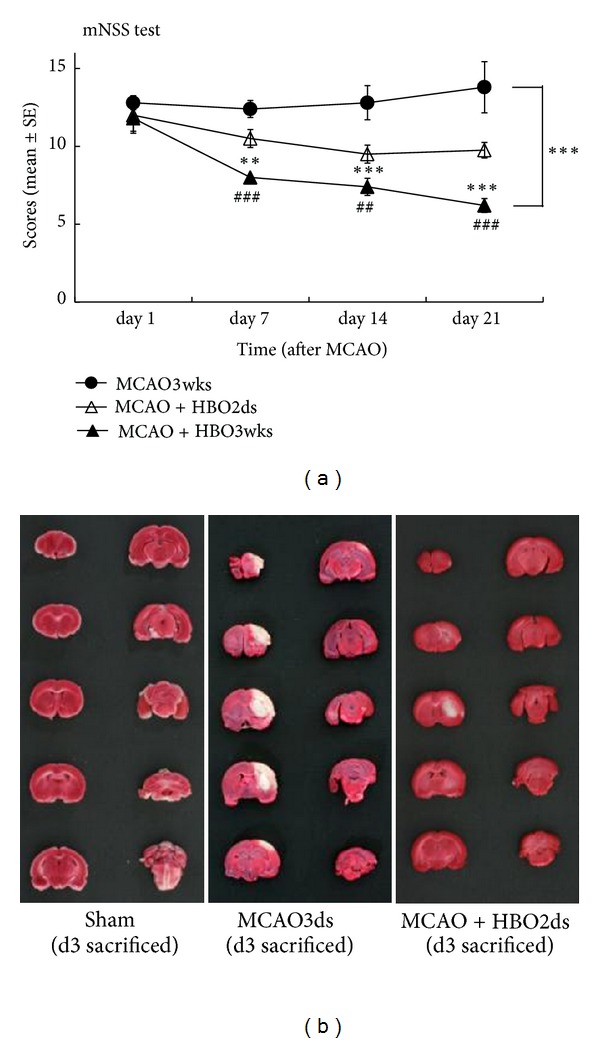
Long course HBO improved functional outcome and decreased infarction size. (a) Behavior tests showed HBO significantly improved function outcome with dose-dependent effect. The mNSS in groups received HBO therapies is significantly less than that in control group ( ***P < 0.001). The declining curve of mNSS in the HBO3wks group became more obviously after day 14. (b) Infarcted area shown on TTC staining (white color) was prominent in the MCAO group but decreased in HBO treated groups.

HBO therapy outcomes in rats were evaluated using the modified neurological severity score (mNSS). A lower score indicated rats had less neurological defects from MCAO, presented with more improved outcome by HBO therapy. Behavior tests showed rats had significantly improved functional outcome when receiving longer repetitive HBO therapy (*P* < 0.001). Despite there only being two days of HBO therapy in the HBO2ds group, rats still showed functional improvement in the following days, with expressing declining curve of mNSS, until day 14 (*P* < 0.01). And the declining curve of mNSS in the HBO3wks group became more obvious after day 14 (*P* < 0.01) (Figure 2(a)). TTC staining showed the ischemic area on the rat's brain tissue. More white color change over brain tissue was found in the MCAO group, which was correlated with more ischemic injury, compared with other groups (Figure 2(b)). Behavior tests were evaluated with mNSS: MCAO3wks, rats sustained MCAO, but without HBO therapy; HBO2ds, rats sustained MCAO, but with two days HBO therapy; HBO3wks, rats sustained MCAO, but with repetitive HBO therapy for three weeks. The infarcted area showed TTC staining (white color) was prominent in the MCAO group, but decreased in HBO treated groups. This indicated that HBO might attenuate cerebral ischemic injury in rats (Figure 2).

### 3.2. HBO Improved BMSCs Migration to Brain

**Figure 3:**
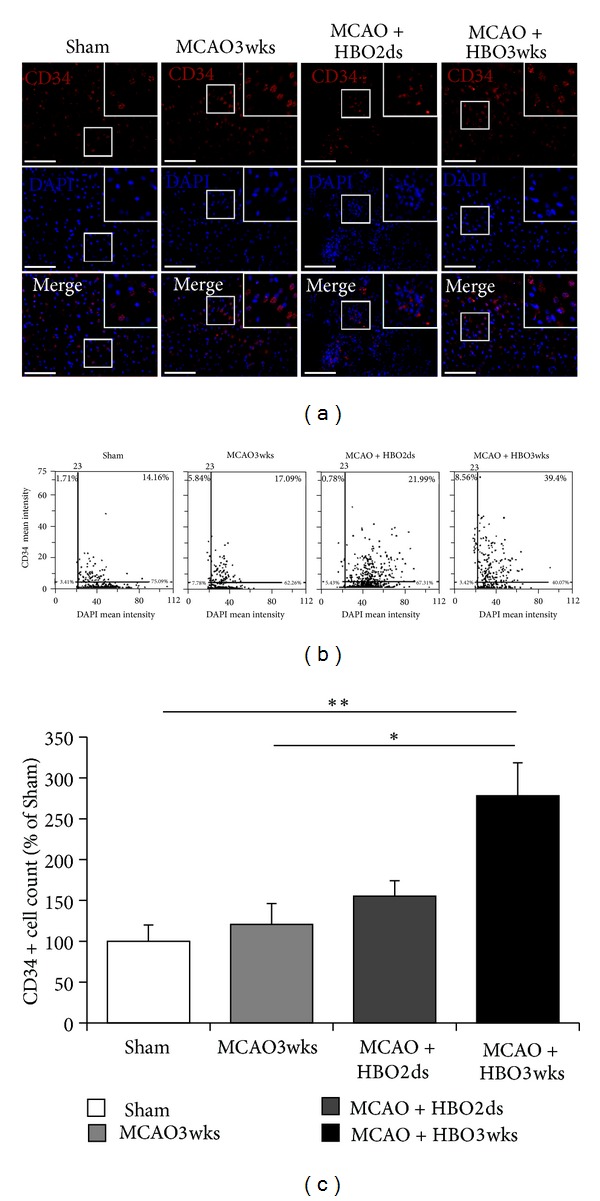
Long course HBO improved BMSCs migration to brain. (a) Demonstration of CD34-DAPI double staining showed presentation of BMSCs after brain ischemia. (b) Representative image of tissue cytometry using TissueQuest software. CD-34 positive cells and DAPI positive cells were counted and the signal intensity was quantified. (c) The amount of double positive cells with CD34 and DAPI in the ischemic boundary was recorded as the percentage of CD34 positive cells in all cells. The difference was significant, as compared with MCAO3wks group and HBO3wks group ( *P < 0.05). The difference was more significant, as compared with Sham group and HBO3wks group ( **P < 0.01).

CD34-DAPI double staining showed the presentation of BMSCs in brain tissue. The number of CD34-DAPI double staining cells was higher in the HBO3wks group, as compared with the shame group, MCAO3wks group, and HBO2ds group (Figure 3(a)). This indicated that rats would recruit BMSCs to brain after acute stroke injury, without HBO therapy. However, the recruited amount of BMSCs was not enough. HBO therapy promotes migration of BMSCs to brain after focal ischemic injury. Longer duration and repetitive HBO would enhance increased BMSCs migration (Figures 3(b) and 3(c)). Migrated BMSC, presenting with double staining of CD34 and DAPI, were found in the MCAO3wks group, HBO2ds group, and HBO3wks group. And there were increased numbers of purple cells (double staining with CD34, red color, and DAPI, blue color) in the HBO3wks group. Representative images of tissue cytometry using TissueQuest software are shown in Figure 3(b). CD-34 positive cells and DAPI positive cells were counted and the signal intensity was quantified. The number of double staining cells with CD34 and DAPI was 14.16% in the Sham group, 17.09% in the MCAO3wks group, 21.99% in the HBO2ds group, and 39.4% in the HBO3wks group, respectively. The amount of double positive cells with CD34 and DAPI in the ischemic boundary was recorded as the percentage of CD34 positive cells in all cells. Data were presented as mean± standard error of the mean (SEM). The difference was significant as compared with MCAO3wks group and HBO3wks group (*P* < 0.05). The difference was even more significant as compared with Sham group and HBO3wks group (*P* < 0.01).

### 3.3. HBO Increased MCAO-Induced Neurogenesis

**Figure 4:**
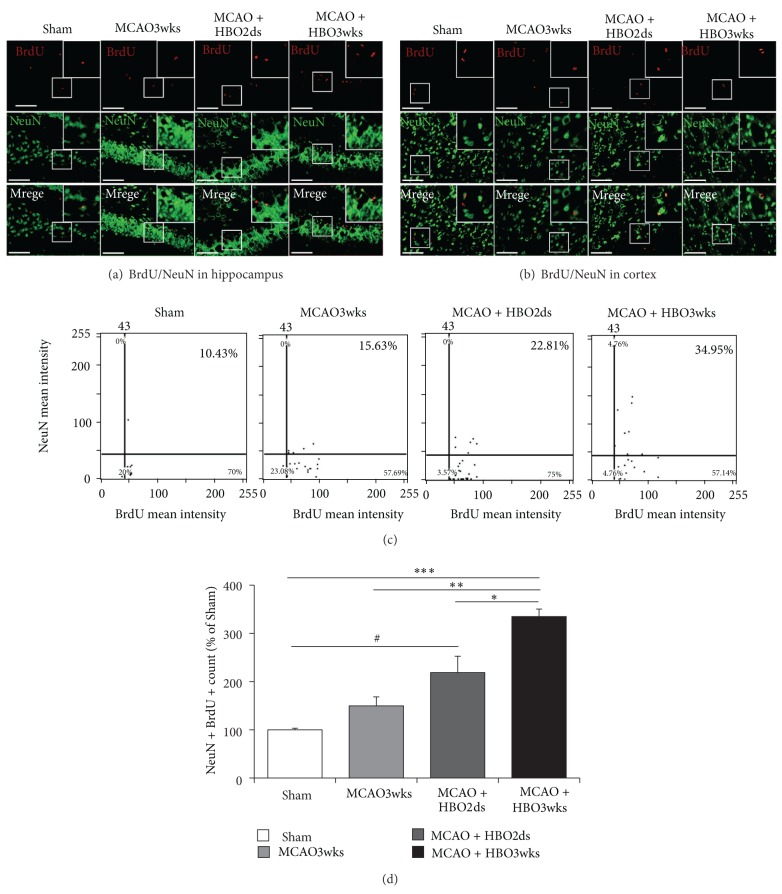
Long course HBO increased neurogenesis. ((a) and (b)) BrdU-NeuN double staining showed that there were significantly more newly forming neurons in the ischemic boundary area (perilesioned cortex (Figure 4(b)) and hippocampus (Figure 4(a))) of HBO3wks rats than HBO2ds rats. (c) Representative images of tissue cytometry using TissueQuest software. BrdU-NeuN positive cells were counted and the signal intensity was quantified. The intensity of co-staining with BrdU and Neu-N was more prominent in HBO3wks group. The difference was significant, as compared with MCAO3wks group and HBO3wks group ( **P < 0.01). There was still significant difference between HBO2ds group and HBO3wks group ( *P < 0.05). The difference was even more significant as compared with Sham group and HBO3wks group ( ***P < 0.001).

BrdU and Neu-N double staining cells showed the number of new proliferation of neuronal cells in the perilesioned cortex and hippocampus (Figures 4(a) and 4(b)). The BrdU positive cells presented with a red color and the Neu-N positive cells were presenting with a green color. The double staining of BrdU and NeuN cells would present with a yellow color. There were more yellow-colored cells (indicating new proliferated neuronal cells) found in the HBO2ds group and HBO3wks group. However, the number of double staining cells (yellow color) was higher in the HBO3wks group. This feature indicated HBO improved neurogenesis, but longer and repetitive HBO course would induce a greater degree of neurogenesis.

BrdU-NeuN double staining showed that there were significantly more newly formed neurons (cells with yellow color) in the ischemic boundary area of hippocampus (Figure 4(a)) and perilesioned cortex (Figure 4(b)). There were more BrdU and NeuN double staining cells in the HBO3wk group.  Figure 4(c) showed the representative image of tissue cytometry using TissueQuest software. BrdU positive cells and Neu-N positive cells were counted and the signal intensity was quantified. The number of double staining cells with BrdU and Neu-N was 10.43% in the Sham group, 15.63% in MCAO3wks group, 22.81% in HBO2ds group, and 34.95% in HBO3wks group, individually. The amount of double staining positive cells with BrdU and Neu-N in the ischemic boundary was recorded as the percentage of BrdU positive cells in all cells. Data were presented as mean± standard error of the mean (SEM). The difference was significant, as compared with the MCAO3wks group and HBO3wks group (*P* < 0.01). There was still a difference between the HBO2ds group and HBO3wks group (*P* < 0.05). The difference was more significant, as compared with the Sham group and HBO3wks group (*P* < 0.001).

### 3.4. HBO Increased MCAO-Induced Gliosis

**Figure 5:**
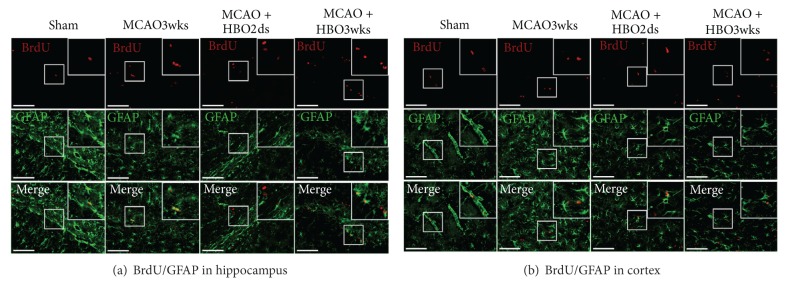
Long course HBO increased gliosis. ((a) and (b)) Demonstration of double staining showed that there were significantly more newly forming or reactive glia in the ischemic boundary area (dentate gyrus (a) and cortex (b)) of HBO3wks rats than HBO2ds rats. The intensity of co-staining with BrdU and Neu-N was more prominent in HBO3wks group.

BrdU and GFAP double staining cells showed the number of new proliferations of glial cells in the dentate gyrus and perilesioned cortex (Figures 5(a) and 5(b)). The BrdU positive cells were presented with red color and the GFAP positive cells were presented as a green color. The double staining of BrdU and GFAP cells would present as a yellow color. There were yellow-colored cells (indicating new proliferated glial cells) found in the MCAO3wks group (24.61%), HBO2ds group (22%), and HBO3wks (30.93%) group. However, the number of double staining cells (yellow color) seemed higher in the HBO3wks group. This suggests that longer and repetitive HBO course would induce a greater degree of gliosis. Demonstrated figures of BrdU-GFAP double staining cells showed that there were more newly forming or reactive glia in the ischemic boundary area (dentate gyrus (Figure 5(a)) and cortex (Figure 5(b))). And there were more double staining cells (yellow colored cells) in the HBO3wks group. The amount of BrdU and GFAP double staining positive cells in the ischemic boundary was recorded as the percentage of BrdU positive cells in all cells. Data were presented as mean ± standard error of the mean (SEM). Despite there being more double staining cells found in the HBO3wks group, there was no statistical difference with regards to the HBO3wks group, as compared with other groups.

### 3.5. HBO Reduced MCAO-Induced Inflammation in the Acute Phase

**Figure 6:**
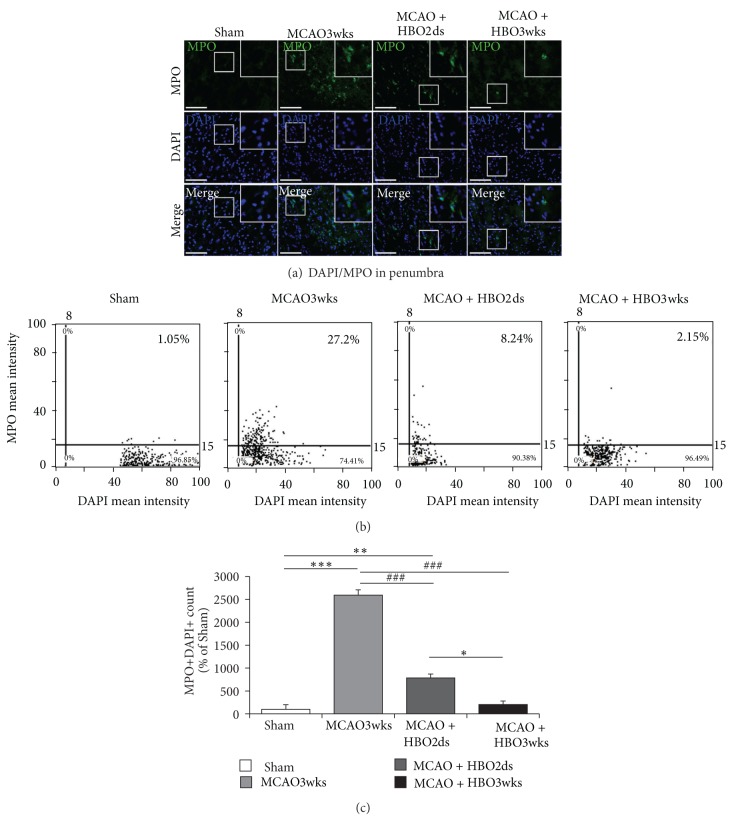
Long course HBO reduced inflammation. (a) MPO staining in penumbra striatum showed there was lower MPO expression in HBO2ds and HBO3wks group. (b) Representative images of tissue cytometry using TissueQuest software. MPO positive cells and DAPI positive cells were counted and the signal intensities were quantified. (c) The amount of double positive cells with MPO and DAPI in the ischemic boundary was recorded as the percentage of MPO positive cells in all cells. The difference was significant, as compared with HBO2ds group and HBO3wks group ( *P < 0.05). The difference was more significant, as compared with MCAO3wks group and HBO3wks group ( ***P < 0.001).

Acute inflammation in the acute phase of cerebral infarction would recruit neutrophils, depositing in the vessels of ischemic boundary area of brain, and caused further brain tissue injury. MPO expression was evaluated for degree of acute inflammation and MPO staining showed the presentation of MPO in penumbra striatum (Figure 6(a)). There was found with decreased MPO expression in the HBO2ds group (8.24%) and HBO3wks group (2.15%), compared with MCAO3wks group (27.2%) (Figures 6(b) and 6(c)). It indicated that HBO treatment would reduce MPO expression, representing with attenuating acute inflammation. Longer and repetitive HBO therapy seemed to have a greater effect in attenuating inflammation.

### 3.6. HBO Increased MCAO-Induced Neurotrophic Factor Level

**Figure 7:**
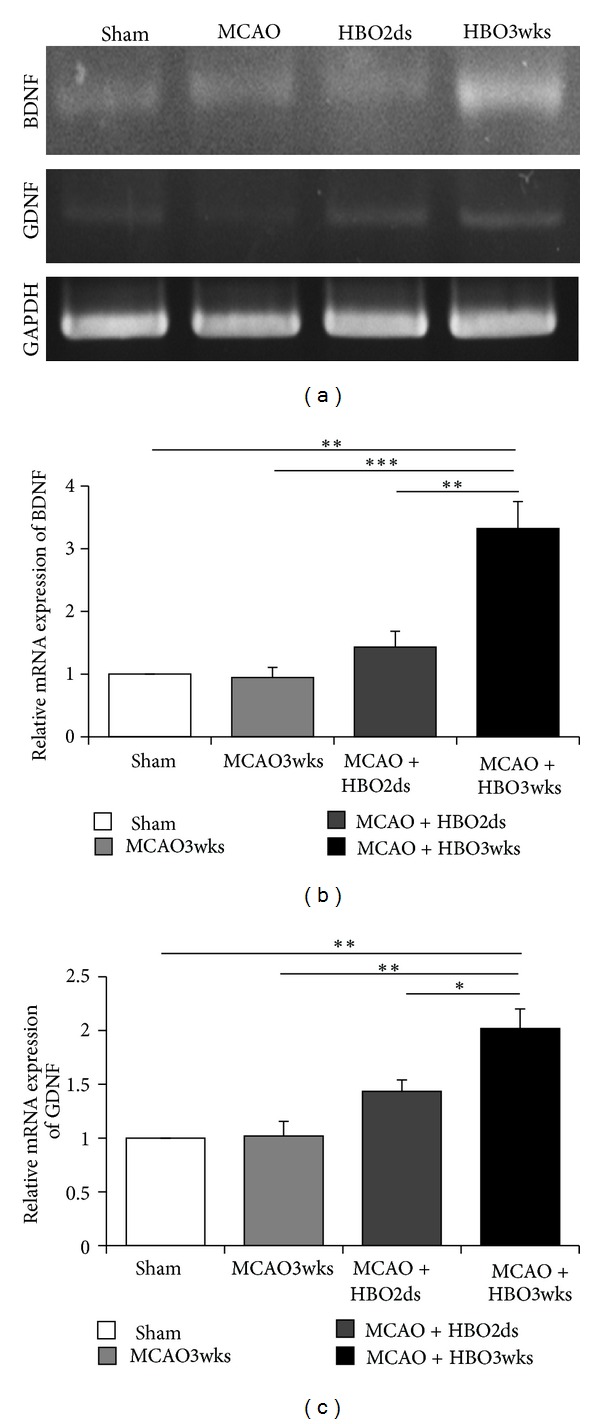
Long course HBO increased neurotrophic factor level. (a) The mRNA levels of BDNF and GDNF showed that mRNA expression levels were increased in HBO treatment group. ((b) and (c)) Expression levels of BDNF and GDNF were significantly increased in the HBO3wks group (*P* < 0.05).

Expression of neurotrophic factor, including BDNF and GDNF, was evaluated by using reverse transcription PCR (RT-PCR). Neurotrophic factors might provide paracrine effect to cells nearby and promote cell proliferation. The mRNA expression of BDNF and GDNF showed that there were BDNF and GDNF production in the MCAO rats and MCAO rats treated with HBO. However, higher expression of BDNF and GDNF was found in the HBO3wks group (Figure 7(a)). There was more BDNF and GDNF production in the HBO3wks group (individually increasing 3.32- and 2.02-fold than Sham), compared with HBO2ds group (individually increasing 1.43- and 1.43-fold than Sham) and MCAO3wks group (individually increasing 0.94- and 1.02-fold than Sham) (Figures 7(b) and 7(c)). The results indicated that longer and repetitive HBO therapy promoted more neurotrophic factor production, including BDNF and GDNF. 

## 4. Discussion

Stroke therapy is an important topic because strokes may result in death or disability, change patients daily life quality, and significantly increase social-economic costs. Ischemic stroke is characterized by cerebral artery occlusion, causing regional cerebral flow reduction or interruption. Previous study showed that hyperbaric oxygen treatment could attenuate ischemic injuries in adult rats [18].

HBO therapy enhanced brain tissue oxygenation during treatment. However, after the termination of HBO treatment and returning to normal oxygenation, the body was temporarily in a relative hypoxic status. This hyperoxia and then turning with relative hypoxia, naming oxygen cycling, would lead to hypoxia inducing factor-1*α* (HIF-1*α*) production [19, 20]. The preconditional hypoxia would decrease ischemic stroke related injury [21]. Recent studies also showed oxygen cycling helped in traumatic brain injury [19] and stem cell therapy for myocardial infarction [22]. The above studies suggested that oxygen cycling may attenuate ischemic injuries.

In our study, 3 weeks of oxygen cycling treatment course for rats with MCAO had better functional outcome compared with rats treated with two-day hyperbaric oxygen treatment course. It presented with lower mNSS, decreased infarction size on brain slice, and correlated with decreased area of TTC staining. There were several beneficial effects in HBO treated stroke, including with reduction of blood-brain barrier permeability, brain edema [22], and attenuation of inflammation [23]. HBO may also attenuate hydroxyl radical production and glutamate release and therefore decrease brain damage [24].

Despite hyperbaric oxygen might cause oxidative stress, activate reactive oxygen species (ROS) (meaning production of reactive oxygen free radical), was thought harmful to brain. Oxygen-activated reactive oxygen species (ROS) has been shown to activate nitric oxide synthase (NOS) [25], promoting NO generation. NO was well known as a potent vasodilator and was crucial in attenuating platelet aggregation, superoxide production, and modulating microvascular permeability [22, 26–28]. Previous study found HBO increases nitric oxide levels in perivascular tissues via stimulation of nitric oxide synthase (NOS) [29]. Furthermore, HBO also stimulate NOS in bone marrow. Bone marrow stem cells will be more proliferated, more easily moving out of bone marrow (mobilization), and homing to ischemic lesions (migration) after HBO treatment [6].

HBO could stimulate vasculogenic stem cell mobilization from bone marrow of diabetics and more cells are recruited to skinwounds to help healing [30, 31]. And in traumatic brain injury, HBO might stimulate neurogenesis. Bone marrow stem cells might be recruited to brain after injury [19]. Despite previous studies about HBO treatment in cerebral infarction in rats showing that bone marrow stem cells were recruited to ischemic brain, most of these studies used short HBO cycling courses. In our study, three weeks of oxygen cycling treatment course induced more BMSCs mobilization to the brain than a two days course. The CD34+ cells were found in ischemic boundary area. This is corresponding with migration of BMSCs to the ischemic brain tissue [32, 33]. This data suggested the dose dependent effect of HBO treatment on homing of BMSC to ischemic brain tissue. Under HBO therapy, BMSCs were more prominent in the group 3wks, than the group 2ds. This data showed that repetitive HBO therapy activated more BMSCs mobilization from bone marrow, then migration to the brain.

In this study, repetitive and longer HBO treatment increased more BMSCs mobilization to the ischemic brain. It was known that BMSCs traffic to the ischemic tissues was relaed to the interaction with stromal-cell-derived factor-1*α* (SDF-1*α*) and chemokine receptor 4 (CXCR4) [34, 35]. In the rats with ischemic stroke, SDF-1 level is significantly increased in the injured hemisphere compared to the uninjured hemisphere [36]. In ischemic stroke patients, It was also found with increased SDF-1*α* [37]. The secretion of SDF-1*α* may act as a chemoattractant to facilitate the homing of circulating CXCR4 positive cells (such as BMSCs) [38] and then help injured tissue with cell repairment. Therefore, further studies about HBO, SDF-1*α*, and CXCR4 are still needed.

The homing by BMSCs is very important in regenerative therapy, not only in stroke treatment, but also in other CNS diseases. BMSCs transplantation in rats were found to attenuate neuronal injury in ischemic stroke [7], spinal cord injury [8], and traumatic brain injury [9]. In the degenerative CNS disease such as Alzheimer's disease (AD), subcutaneous injection with G-CSF would increase BMSCs mobilization to brain to rescue the lesions [39]. In this study, we provided another aspect for HBO treated stroke in the point of view of BMSCs rescue. The homing of BMSCs to the ischemic boundary of rat brain, stimulated by HBO, would promote neural plasticity, stimulate production of growth factor or cytokine, and attenuate inflammation [40].

BrdU-labeled cells with expressing Neu-N (unique to neuron and axon) were present in HBO treated MCAO rats. The expressing level was more prominent in the group of HBO3wks, compared with the group of HBO2ds. This indicated that longer oxygen cycling had better protection of ischemic rats, far from neural loss, by increasing neural cell numbers. Furthermore, those migrated BMSCs, more promoted by HBO, might have cell transdifferentiation [41] or help axon remodeling [42]; they might also stimulate mRNA transcription of growth factor and cytokine, which would activate neural stem cells (already housing in the dentate gyrus and subventricular zone) [43, 44] to proliferate and migrate.

In the study, BrdU-labeled cells with expressing GFAP (fairly to glial lineage) were present in HBO treated MCAO rats. The expressing level was more prominent in the group of HBO3wks, compared with the group of HBO2ds. This indicated that there was more degree of gliosis in the group of HBO3wks. Recent studies have emphasized the dual effects of gliosis (both detrimental and beneficial in neuroprotection and functional recovery) [45]. Time and signaling molecule for gliosis were referring to its final fate [46]. Corresponding to final functional improvement in rats, we presumed that longer oxygen cycling might help overcoming detrimental effect of gliosis and donating its beneficial effect, by regulating inflammation and influencing trophic factor production.

In this study, neurotrophic factors such as BDNF, NGF, and GDNF were evaluated. Under oxygen cycling, the level of BDNF, NGF, and GDNF were increased compared with control group. And there was more BDNF and NGF production in the group HBO3wks, compared to the group HBO2ds. A concept of “entire protection of neurovascular unit” in cerebral infarction treatment has been proposed, which suggested the protection of neurons alone might not work and emphasized the interaction between neurons, glia, and the cerebral endothelium [47]. Cerebral endothelium was the major source of BDNF. Brain-derived endothelial medicated paracrine and autocrine via BDNF and NGF [48]. More BDNF production indicated that cerebral endothelium was with less damage and getting recovery [12] and perhaps contributed to increased cell numbers from transdifferentiation of migrated BMSCs or secretion of trophic factor by grafted BMSCs [49].

Inflammation is a defense response against the insults that limit injury and remove noxious agents [50]. Ischemic stroke is associated with acute and prolonged inflammatory process characterized by the activation of resident glial cells, production of inflammatory cytokine, and leukocyte and monocyte infiltration in the brain [51]. Neutrophil recruitment may contribute to microvascular occlusion, releasing proinflammatory cytokine, and then further cause damage. In this point of view, MPO activity, presenting neutrophil activity, was evaluated in this study. The result showed that there was lower MPO activity in the group HBO3wks. It indicated longer oxygen cycling would attenuate inflammation. The result was also correspondent with previous study, which showed that HBO treatment might reduce local MPO activity, neutrophils infiltration, and infarction volume and thus enhance functional outcome for rats with focal ischemia [19, 51].

## 5. Conclusion

HBO therapy might attenuate inflammation in rat with ischemic stroke. It might promote more BMSCs production, mobilization, and migration to ischemic brain. Those BMSCs stimulated neurogenesis and gliosis. This longer oxygen cycling (treated stroke by HBO for repetitive schedules for 3 weeks) therapy orchestrated gliosis and trophic factor production (BDNF, NGF, and GDNF) and decreased harmful effects by neutrophils in the early phase.

## Figures and Tables

**Table 1:** Detail description of the items forming the modified neurological severity score (mNSS).

Motor tests	
Raising rat by tail (normal = 0, maximum = 3)	(3)
Flexion of forelimb	1
Flexion of hindlimb	1
Head moved >10 degree limb vertical axis within 30 s	1
Placing rat on floor (normal = 0, maximum = 3)	(3)
Normal walk	0
Inability to walk straight	1
Circling toward the paretic side	2
Falling down to paretic side	3
Sensory tests (normal = 0, maximum = 2)	(2)
Placing test (visual and tactile test)	1
Proprioceptive test (deep sensation, pushing paw against table edge to stimulate limb muscles)	1
Beam balance tests (normal = 0, maximum = 6)	(6)
Balance with steady posture	0
Grasps side of beam	1
Huging beam and 1 limb falling down from beam	2
Huging beam and 2 limbs falling down from beam, or spins on beam (>60 s)	3
Attempting to balance on beam but falling off (>40 s)	4
Attempting to balance on beam but falling off (>20 s)	5
Falling off; no attempt to balance or hang on the beam (<20 s)	6
Reflex absence and abnormal movements (normal = 0, maximum = 4)	(4)
Pinna reflex (head shaken when auditory meatus is touched)	1
Corneal reflex (eyes blink when cornea is lightly touched with cotton)	1
Startle reflex (motor response to brief noise from clapping hands)	1
Seizures, myoclonus, myodystony	1
Maximum points	(18)

One point is given for an absent reflex tested or for the animal's inability to perform a task: 1–6 mild injury, 7–12 moderate injury, and 13–18 severe injury	
